# Differing Visual Behavior Between Inexperienced and Experienced Critical Care Nurses While Using a Closed-Loop Ventilation System—A Prospective Observational Study

**DOI:** 10.3389/fmed.2021.681321

**Published:** 2021-09-08

**Authors:** Philipp K. Buehler, Anique Herling, Nadine Bienefeld, Stephanie Klinzing, Stephan Wegner, Pedro David Wendel Garcia, Michael Karbach, Quentin Lohmeyer, Elisabeth Schaubmayr, Reto A. Schuepbach, Daniel A. Hofmaenner

**Affiliations:** ^1^Institute of Intensive Care Medicine, University Hospital Zurich, Zurich, Switzerland; ^2^Department of Management, Technology, and Economics, Work & Organizational Psychology, ETH Zurich, Zurich, Switzerland; ^3^Department of Mechanical and Process Engineering, ETH Zurich, Zurich, Switzerland

**Keywords:** eye-tracking, user interfaces, closed-loop ventilation, monitoring, visual behavior

## Abstract

**Introduction:** Closed-loop ventilation modes are increasingly being used in intensive care units to ensure more automaticity. Little is known about the visual behavior of health professionals using these ventilation modes. The aim of this study was to analyze gaze patterns of intensive care nurses while ventilating a patient in the closed-loop mode with Intellivent adaptive support ventilation® (I-ASV) and to compare inexperienced with experienced nurses.

**Materials and Methods:** Intensive care nurses underwent eye-tracking during daily care of a patient ventilated in the closed-loop ventilation mode. Five specific areas of interest were predefined (ventilator settings, ventilation curves, numeric values, oxygenation Intellivent, ventilation Intellivent). The main independent variable and primary outcome was dwell time. Secondary outcomes were revisits, average fixation time, first fixation and fixation count on areas of interest in a targeted tracking-time of 60 min. Gaze patterns were compared between I-ASV inexperienced (*n* = 12) and experienced (*n* = 16) nurses.

**Results:** In total, 28 participants were included. Overall, dwell time was longer for ventilator settings and numeric values compared to the other areas of interest. Similar results could be obtained for the secondary outcomes. Visual fixation of oxygenation Intellivent and ventilation Intellivent was low. However, dwell time, average fixation time and first fixation on oxygenation Intellivent were longer in experienced compared to inexperienced intensive care nurses.

**Discussion:** Gaze patterns of intensive care nurses were mainly focused on numeric values and settings. Areas of interest related to traditional mechanical ventilation retain high significance for intensive care nurses, despite use of closed-loop mode. More visual attention to oxygenation Intellivent and ventilation Intellivent in experienced nurses implies more routine and familiarity with closed-loop modes in this group. The findings imply the need for constant training and education with new tools in critical care, especially for inexperienced professionals.

## Introduction

Patients in the intensive care unit (ICU) are particularly vulnerable to harm due to their complex clinical history and critical condition. Owing to a high number of machine user interfaces, challenging and often time-critical processes, the management of critically ill patients involves a high risk of error. Unintentional human errors and lack of situation awareness are among the leading causes of adverse events, not only in the medical setting (1–12). Unintentional errors can be classified into four main sources: slip, lapse, mistake and violations (13). Furthermore, a distinction is made between systemic and individual causes of error (12). Individual causes include distraction, inattention, forgetfulness, motivational deficits, and lack of awareness. Despite decades of research on human factors and sociotechnical system design, in practice the influence of human-environmental interaction (e.g., use of technical devices) is often neglected and could be contributing to individual errors. Increasingly, machines with complex control circuits are challenging human receptiveness. In intensive care medicine, the understanding of human-machine interactions is of particular importance in preventing adverse events (14). Inadequate information processing with respect to monitoring devices may contribute to individual errors leading to impaired patient outcomes. To date, it is largely unclear how user interfaces and specific program modes of technical devices are cognitively processed by specialized ICU nurses, despite some past research on graphical displays and situation awareness (11, 15). More knowledge about human-machine interactions in intensive care medicine is required (9). One example of the emerging role of technical systems in the ICU are closed-loop ventilation modes. In contrast to conventional volume- or pressure-controlled modes, the inherent automaticity of closed-loop modes is reminiscent of autopilot modes in airplanes. Closed-loop ventilation modes operate through an inherent feedback mechanism breath-by-breath. Based on constant measurements (i.e., peripheral oxygen saturation and end-tidal carbon dioxide) and algorithms, these modes automatically adjust the fraction of inhaled oxygen (FiO_2)_, the positive end-expiratory pressure (PEEP) and minute ventilation (16, 17).

When ICU patients are ventilated with closed-loop modes, the specific role allocation and associated tasks of the nurses responsible shift from active, manual machine handling to a rather machine-supervisory role (18). However, it is currently unknown to what extent the transition toward a supervisory role, with its inherent change in the type and assessment of information (19) presented by the new closed-loop ventilation modes, has occurred. Eye-tracking is a tool that enables monitoring of gaze patterns and visual attention. It has been used to analyze various professional scenarios in the medical field, including ICUs (20–32), and might be beneficial in gaining a more profound understanding of the operation of closed-loop ventilation modes.

The aim of this study was thus to analyze gaze patterns of ICU nurses using eye-tracking while ventilating a patient in the closed-loop mode Intellivent adaptive support ventilation (I-ASV)® and to compare the patterns of inexperienced with those of experienced ICU nurses.

## Materials and Methods

### Ethics

The Local Ethics Committee (Kantonale Ethikkommission Zurich BASEC ID REQ 2017-00798) approved the study protocol, guaranteeing accordance with the declaration of Helsinki. Written informed consent was given by all participating ICU nurses and the patients involved, or the patients' legal representatives in cases of incapacity of judgement.

### Study Design and Study Population

This was a prospective, observational, real-life eye-tracking study conducted at the ICU of the University Hospital Zurich (Zurich, Switzerland). The interdisciplinary ICU treats about 4,500 patients per year in 64 ICU beds. All specialized ICU nurses working in the ICU were eligible for participation, provided there were no exclusion criteria. Exclusion criteria were visual disturbances (lack of stereoscopic vision, monocular vision and achromatopsia) or withheld informed consent. The respirators used were “Hamilton S1®” respirators (Hamilton Medical Company, Bonaduz, Switzerland). Independently of this study, all nurses underwent a standardized training program in Intellivent adaptive support ventilation (I-ASV, Hamilton Medical Company, Bonaduz, Switzerland) before bedside application of the closed-loop ventilation mode. During the first year of I-ASV application after professional training, the ICU nurses are constantly supervised by senior/teaching nurses while ventilating their patients, whereas after this period they work without supervision. Thus, for the design of this study, nurses who had worked <1 year with I-ASV were considered inexperienced, whereas nurses who had worked for more than 1 year with I-ASV were considered experienced. Participation in this study was free of charge and voluntary. If a calibration of the eye-tracker was possible, the participant was included. All recordings were performed in the early afternoon in order to avoid biases due to the regular morning rounds with the treating physicians or due to nightshifts, which might impair standardization of data. Further, all recordings were scheduled in order that they did not coincide with special circumstances such as interventions or patient transports.

For study purposes, participating nurses were responsible for one patient. All patients, with various medical conditions, were invasively mechanically ventilated in I-ASV. Patients were only included if the presumed duration of mechanical ventilation was longer than 24 h. Short-term postoperative patients were not included. Patients with severe acute respiratory distress syndrome (ARDS) were not eligible (in the study center, it is a physician's task to adjust ventilator settings in this patient collective). No patient was intubated only for the purposes of this study. Non-intubated patients were not eligible for participation.

### Study Protocol

Prior to the recordings, demographics and data with possible influence on eye-tracking, such as workload of participants were gathered (33). To avoid biases, no information concerning the aim of the study was provided to the participants. In a questionnaire using validated scales, a participant assessment regarding I-ASV performance and effort expectancy, anxiety, and social influence was collected (34). After a period of habituation to the eye-tracking device lasting 30 min, and a three-point calibration, participants were asked to perform their daily nursing tasks including patient care, handling of perfusors, application of drugs and ventilating the patient in I-ASV, which is the default respiration mode in the ICU. The targeted tracking time was 60 min per nurse. This tracking time was predefined by the study team to maximize the collection of data, while avoiding unnecessary interruptions (e.g., breaks, relatives coming for a visit etc.) or participant fatigue occurring in more prolonged recordings.

To provide a study setting as close to reality as possible, no advise was given to the participants on how to use and handle their respirator. Participants were free to use the respirator in the way they considered useful and to look at the respirator as often as they wanted.

After the eye-tracker recordings, a post-experiment questionnaire was completed. Using validated scales, workload and subjective stress during the tracking were assessed (33). Concerning I-ASV, the questionnaire collected data including the perceived safety of this mode, whether participants had enough specific knowledge about its use, their intention to continue using it in the future and facilitating conditions for future use of I-ASV (34).

### Data Analysis

To address the aim of the study, only gaze patterns relating to the user interface of the respirator and fixations on the ventilator were analyzed (e.g., adjusting the settings, checking values, using touch panels). All other visual fixations (e.g., on the patient, perfusors, other staff, other devices, etc.) were not subject to analysis and thus excluded.

**Figure 1:**
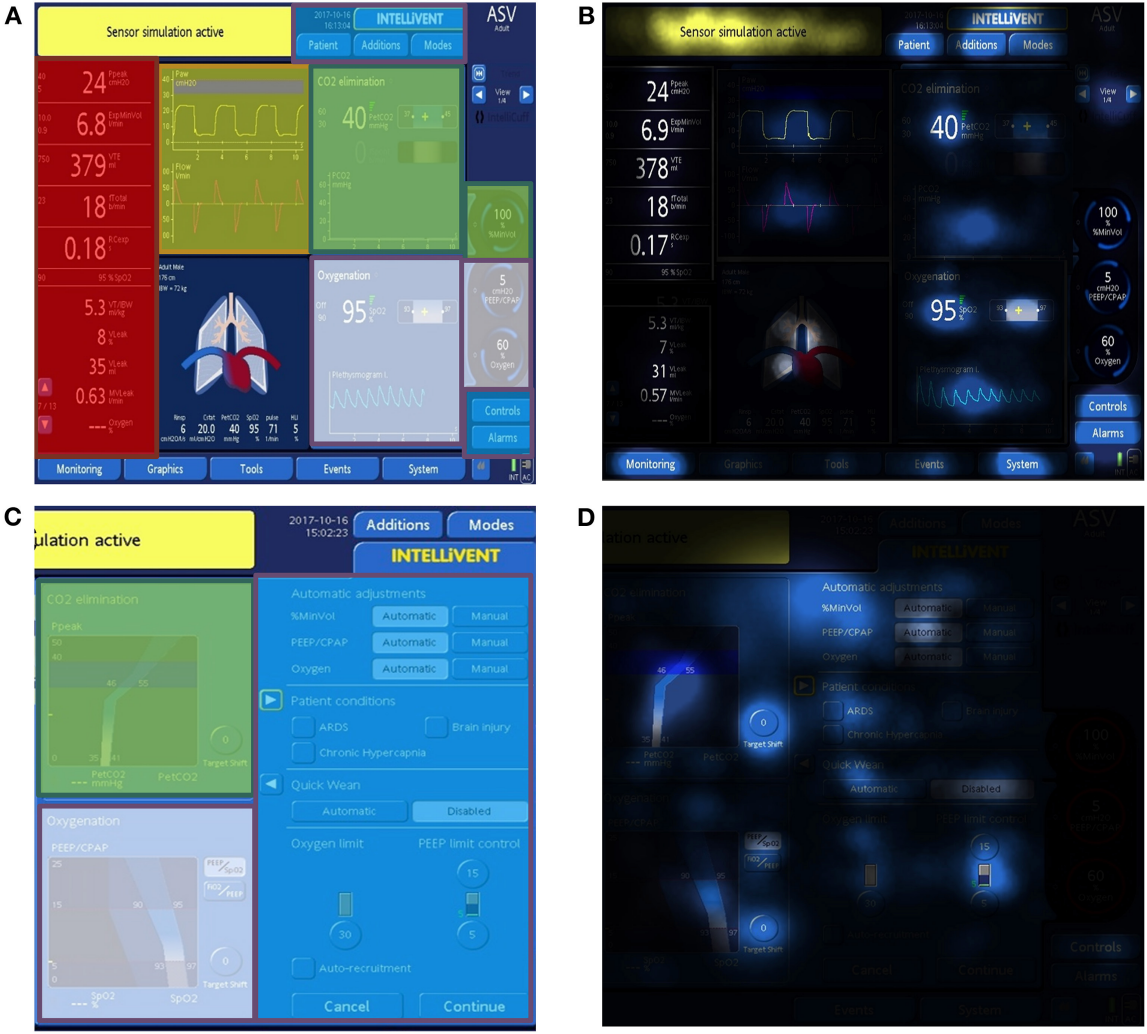
Sample screen panels of the Hamilton Medical S1 respirator in closed-loop ventilation mode. The AOIs settings (blue), ventilation curves (yellow), numeric values (red), oxygenation Intellivent (white) and ventilation Intellivent (green) were pre-defined. The AOIs and the standard display of the screen are visualized in **(A,C)**. **(B,D)** depict the integrated focus maps for dwell time of all participants. In focus maps, more/longer fixations lead to a brighter color. Darker areas indicate fewer fixations.

For our analysis, five areas of interest (AOI; i.e., areas on the ventilator's user interface that were important in addressing the aim of the study) were defined by the study team prior to the recordings (Figures 1A,C). Three AOIs were not related to the closed-loop system I-ASV and included the conventional ventilator settings (including settings for patient data, ventilation modes, alarms), the classic ventilation curves (pressure-, volume-, flow curves) and numeric values on the displays (including e.g., peak pressure, tidal volume, minute volume, respiratory frequency, end-tidal carbon dioxide CO2). The remaining two AOIs were specifically designed to address the use and the program modes of I-ASV. The AOI “oxygenation Intellivent” combined the oxygenation parameters and controlling oxygenation in I-ASV (including Intellivent oxygenation graphics, positive end-expiratory pressure (PEEP), PEEP limits, fraction of inhaled oxygen (FiO2), target shift for oxygenation). The other AOI “ventilation Intellivent” included ventilation parameters and controlling minute volume in I-ASV (including Intellivent ventilation graphics, %minute-volume, target shift for decarboxylation) (Figures 1A,C). All visual fixations on other, irrelevant areas of the respirator (e.g., white space, non-determinable fixations, valves, gauges, tubes) were excluded.

### Primary Outcome

The primary outcome was dwell time (cumulated time spent on an area of interest including fixations, blinks and saccades) for the specific AOIs.

### Secondary Outcomes

Secondary outcomes were revisits (the frequency of revisiting a particular area of interest after gazing at other areas), average fixation time, first fixation (duration of the first fixation of an AOI) and fixation count (the cumulated number of gaze fixations on a particular AOI) to all AOIs.

### Subgroup Analyses

In subgroup analyses, inexperienced nurses (<1 year experience with I-ASV, as described above) were compared with experienced nurses.

### Data Recording

The SMI Eye-tracking Glasses 2 Wireless system (SensoMotoric Instruments, Teltow, Germany) was used. Gaze-tracking was executed at a sampling rate of 60 Hz. Over all distances, the angle of view was measured with an accuracy of 0.5°. The scene video was recorded with a resolution of 960 × 720 pixels at 30 fps. To record audio data, an integrated microphone was used. Eye-tracking data were processed using the SMI BeGaze 3.6 software (SensoMotoric Instruments, Teltow, Germany) and the SMI algorithm for fixation determination. Each ocular fixation during the handling of the respirator was manually assigned to the above-mentioned AOIs.

### Statistics

No power calculation was performed due to the absence of preliminary tests and partial descriptive statistics. Based on valid data from other eye-tracking studies in the critical care setting (24), a participant number of more than 20 was considered adequate. Data were expressed as the median and interquartile range (25th−75th percentile) for continuous variables or as percentages for categorical variables. Discrete variables were compared using the Chi-square or Fisher exact test, as appropriate. Groups of continuous variables were compared by Mann-Whitney U test, owing to the non-parametric data. For multiple comparisons, Friedman's test with Dunn's correction was used. A *p*-value of < 0.05 (two-sided p) was considered statistically significant.

Statistical analysis was performed using SPSS Version 23 (SPSS Science, Chicago, IL, USA) and Graphpad prism 7 (San Diego, CA, USA).

## Results

Of a total of 30 ICU nurses assessed, two could not be included owing to exclusion criteria. The remaining 28 agreed to participate in this study and were divided into two groups (inexperienced and experienced groups), with 12 nurses assigned to the inexperienced group and 16 to the experienced group. Median age was 39.5 years; 86% of all participants were female. Table 1 presents the baseline characteristics of all participants.

**Table 1:** Baseline characteristics of participants.

**Baseline characteristics**		
Age	Years	39.5 (29–45.5)
Sex	Male	4 (14.3%)
	Female	24 (85.7%)
Vision correction	No	17 (60.7%)
	Yes	11 (39.3%)
Professional experience total	Years	18 (5.5–25)
Professional experience ICU	Years	11.5 (3–16.5)
Being rested^*^	(Scale 0–10)	7 (6–8)
Mental workload before tracking^*^	(Scale 0–20)	12.5 (10–14.8)
Physical workload before tracking^*^	(Scale 0–20)	10.5 (8–12.5)
Mental workload during tracking^*^	(Scale 0–20)	12.5 (6.3–14)
Physical workload during tracking^*^	(Scale 0–20)	7.3 (5.5–11)
Subjective stress during tracking^*^	(Scale 0-10)	4 (2–5)

*Data expressed as number (%) or median and interquartile range (IQR). Workload/stress assessed using a numeric scale (where 0 = totally relaxed and 20 = totally stressed)*.

* *marks a subjective and self-assessed characteristic*.

The subjective mental and physical workloads assessed by the validated NASA-TLX scale (33) before and during the recordings were similar across all participants. Subjective stress during tracking was given a median score of 4 points on a numerical rating scale ranging from 0 to 10 (Table 1). No participant was subjectively disturbed by the eye-tracking glasses. No patient emergencies occurred during the recordings and no recordings had to be interrupted or terminated.

Compared to the total tracking time, median fixation of the respirator was 13% and did not differ between the study groups.

**Figure 2:**
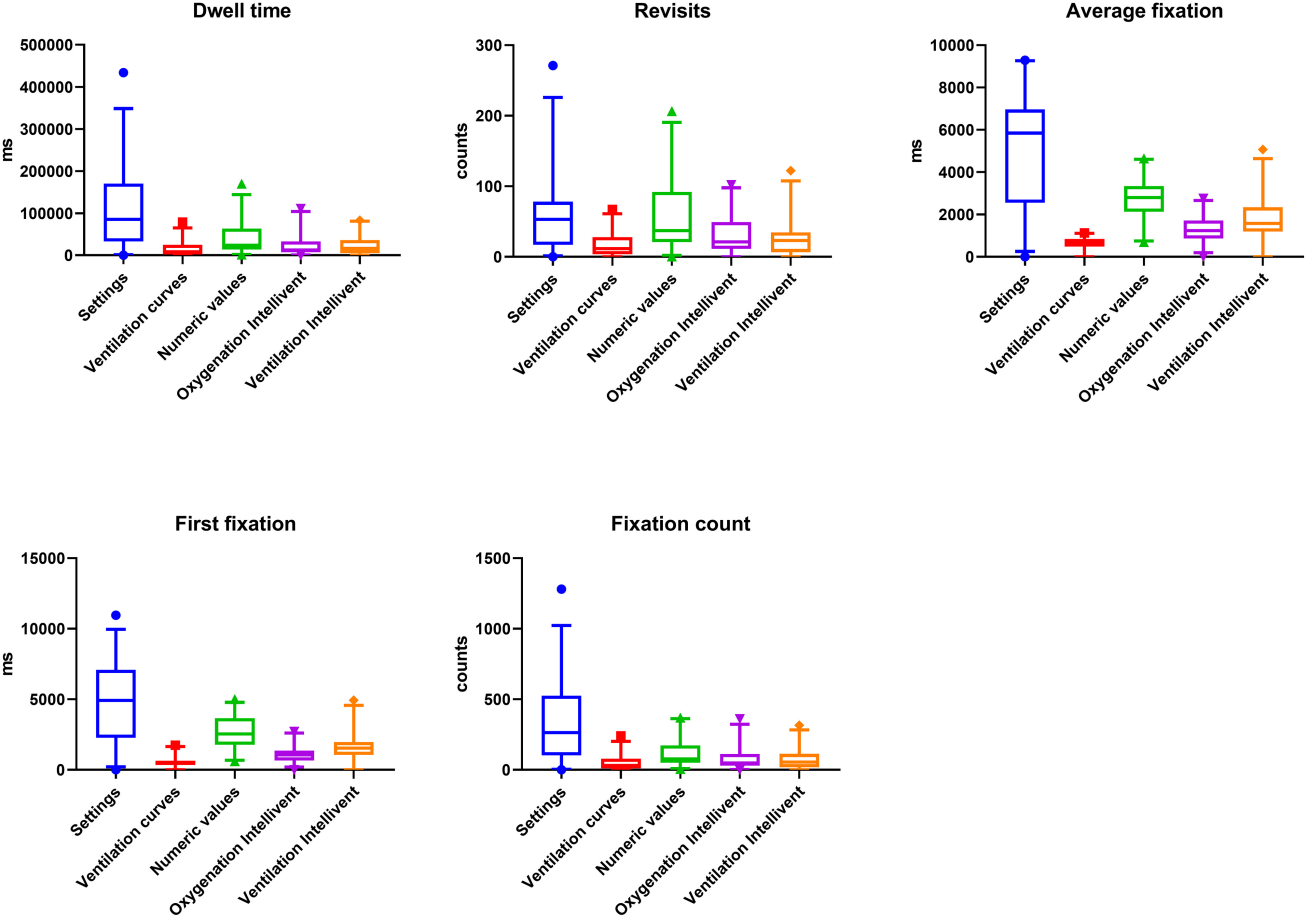
Dwell time, Revisits, Average fixation time, First fixation, and Fixation count for all AOIs across the two groups (inexperienced and experienced). Absolute values and *p*-values for multiple comparisons analyzed by Friedman's test are provided in the Supplementary Tables 1, 2.

Overall, dwell time was significantly prolonged for the settings compared with the other AOIs (Figures 1B,D, 2). Similarly, the number of revisits, the average fixation time, first fixation and fixation count were higher for the settings. Furthermore, there was an increased number of revisits, average fixation time and first fixation for the numeric values compared with the other AIOs (Figure 2).

Overall, visual attention to the AOIs oxygenation Intellivent and ventilation Intellivent were low for all outcome parameters. Visual attention to the ventilation curves was lowest compared with the other AOIs evaluated. The absolute values for dwell time, revisits, average fixation time, first fixation and fixation count are indicated in Supplementary Table 1. *P*-values for multiple comparisons are provided in Supplementary Table 2.

**Figure 3:**
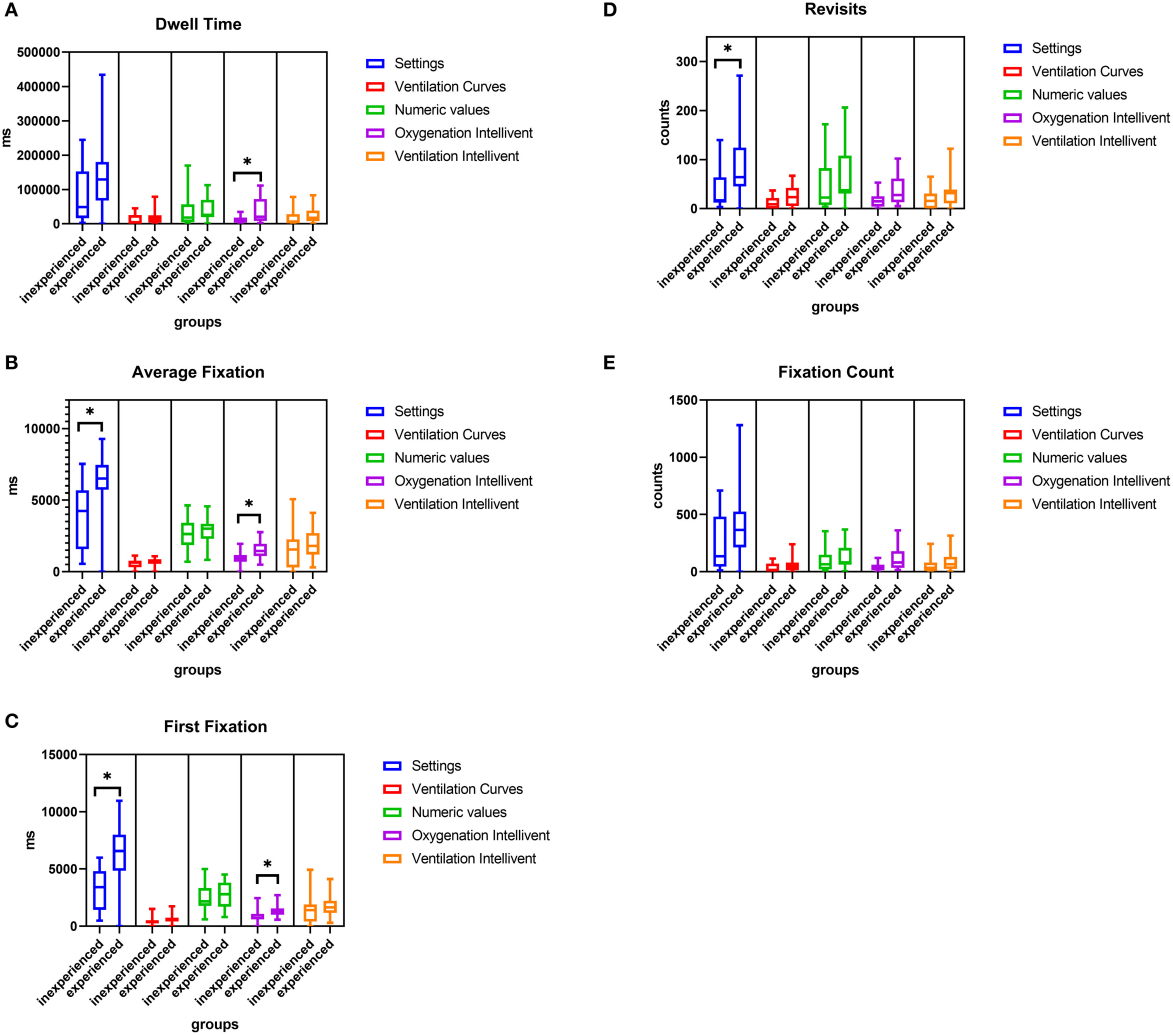
Comparison of Dwell time **(A)**, Average fixation time **(B)**, First fixation **(C)**, Revisits **(D)**, and Fixation count **(E)** for all AOIs for inexperienced and experienced participants. *p < 0.05. Exact *p*-values for all group comparisons are provided in the Supplementary Table 3.

Figure 3 depicts the subgroup analysis for different professional experience.

Dwell time, average fixation time and first fixation on oxygenation Intellivent were significantly higher in experienced participants, and showed a trend toward being elevated for ventilation Intellivent. For the AOI settings, the revisits, average fixation time and first fixation were higher in the experienced group. The *p*-values for all group comparisons are provided in Supplementary Table 3.

Table 2 summarizes data derived from the pre- and post-experiment questionnaires. In particular, it shows that closed loop ventilation is predominantly used on a daily basis. Overall and regardless of previous experience, participants had a positive attitude toward the use of closed-loop ventilation, considered it useful and intended to use it in the future. Subgroup analyses revealed, however, that inexperienced nurses reported significantly higher levels of anxiety toward using I-ASV compared with experienced nurses.

**Table 2:** Pre- and post-experiment questionnaires.

	**Group**		
**Pre-experiment**	**Inexperienced**	**Experienced**	***p*-value**
	**(*n* = 12)**	**(*n* = 16)**	
Professional experience ICU	7.5 (1–15)	14.5 (4.5–18)	0.159
Experience with I-ASV (scale 0–10)^*^	1.25 (0.85–3)	3 (2.75–5)	**0.006**
Years using I-ASV (*n*)	0.9 (0.4–1)	3 (2.25–4)	**0.001**
How often is I-ASV used in everyday practice (scale 0–20)	15 (5.75–17)	15.5 (6.5–19)	0.397
Usefulness of I-ASV (scale 0–7)^*^	5 (4.5–6)	5 (5–6)	0.478
Productivity with I-ASV (scale 0–7)^*^	5 (3–5)	4 (3–6)	0.664
Improved patient care with I-ASV (scale 0–7)^*^	5 (3.5–6)	4.5 (4–5.5)	0.698
Afraid to make mistakes while using I-ASV (scale 0–7)^*^	3 (2.5–5)	1 (1–2)	**0.003**
Intimidation caused by I-ASV (scale 0–7)^*^	3 (1.5–5)	1 (1–2)	**0.008**
**Post-experiment**
I-ASV provided more resources (scale 0–7)^*^	5 (5–6)	4.5 (4–5)	0.195
Had enough knowledge to use I-ASV (scale 0–7)^*^	5 (3–5.5)	6 (5.5–7)	**0.017**
Intention to use I-ASV in the future (scale 0–7)^*^	7 (6–7)	7 (6–7)	0.837
Use of I-ASV is safe (scale 0–7)^*^	3.5 (2–6)	3 (2–4.5)	0.568

*Data expressed as median and interquartile range (IQR)*.

* *marks a subjective and self-assessed characteristic. A higher number represents a higher acceptance of the statement. Groups compared via Mann-Whitney-U test, with < 0.05 considered significant (significant p-values in bold)*.

## Discussion

The aim of this study was to analyze gaze patterns of specialized ICU nurses while their patients were undergoing ventilation in the closed-loop mode Intellivent adaptive support ventilation® (I-ASV) and to compare inexperienced with experienced nurses.

The main results of this study imply that, despite the use of a closed-loop ventilation mode, both inexperienced and experienced ICU nurses' gaze patterns were predominantly linked to conventional control and monitor panels (Figure 2). As an expression of the nurses' visual attention, the dwell time of the two AOIs settings and numeric values was elevated. The importance of these conventional panels was also mirrored in the significantly higher number of revisits. Moreover, the average fixation time on these AOIs was high as well. This finding suggests that possibly necessary altered visual behavior focusing on new monitoring panels, such as oxygenation and ventilation Intellivent, has not yet been adopted across critical care professionals independent of their experience level and should be specifically trained in the future. As a possible explanation for our findings, relevant visual fixation on numeric values (e.g., peak pressure, tidal volume, respiratory frequency) might represent a high degree of familiarity to ICU nurses. The frequent glances at common numbers reflecting respiratory parameters might mentally be easily linked to ventilation strategies such as protective lung ventilation. As such, it seems plausible that nurses frequently focus on numeric values in order to assure lung protection and anticipate possible ventilation adaptions early.

Overall visual attention to oxygenation Intellivent and ventilation Intellivent for the primary and secondary outcomes was markedly lower, despite the use of a closed-loop ventilation mode. Closed-loop ventilation modes such as I-ASV have emerged as new ventilation strategies, but have not been consistently adopted in critical care medicine to date. Our results demonstrate that information displayed by closed-loop ventilation might not be visually presented or mentally processed to a sufficient extent. The frequent visual focus on classic ventilation parameters such as numeric values could also be a sign of a lack of trust in new ventilation modes, especially for less experienced nurses, who reported a significantly higher level of anxiety associated with the use of I-ASV. Furthermore, the question about the subjective safety of I-ASV was answered with a neutral value of 3 on a scale from 1 to 7 (Table 2), which might indicate that some degree of skepticism toward modern ventilation modes still remains. However, the use of a single (albeit validated) scale plus one question assessing the subjective safety of I-ASV in the post-experiment questionnaire makes it difficult to draw a conclusive statement. Further trials should compare the visual behavior of ICU nurses between closed-loop and a conventional mode (e.g., pressure-controlled mode).

The sub-analysis of the two professional experience levels revealed that there were differences in gaze behavior between inexperienced and experienced participants. The dwell time and average fixation time on oxygenation Intellivent were significantly longer in experienced nurses. For the AOI settings, the revisits, average fixation time and first fixation were higher. On the one hand, these differences might mirror the greater importance of these AOIs. Research on performing skills among differently trained groups has shown that experts in particular try to focus their attention on critical areas, which is called “target locking” (35, 36). This concept could also be the reason that the dwell time of the experienced nurses was either significantly increased or at least a trend, especially for the settings and the oxygenation and ventilation Intellivent, which are important AOIs in monitoring patients and their clinical condition. In our opinion, the elevated revisits reflect frequent checking glances among the experienced participants. On the other hand, the findings would also support the hypothesis that greater experience might enhance familiarity and routine with the use of closed-loop systems. In line with this postulation, inexperienced nurses might have felt more intimidated by I-ASV and/or were more afraid of making mistakes than experienced nurses, as reflected by the significantly higher levels of anxiety toward the use of I-ASV. Blind faith in the new technology could also have been the reason why the number of revisits and dwell time for the above-mentioned AOIs among novices was reduced. Moreover, in the post-experiment questionnaire, inexperienced nurses reported a lack of knowledge about I-ASV compared with their more experienced counterparts.

One advantage of closed-loop modes could be the enhanced automaticity with less manual adaptation needed to adhere to lung protective ventilation. This implies that frequent visual focus on the conventional AOIs of ventilator curves or numeric values is probably no longer necessary. However, the use of closed-loop systems requires familiarity, ongoing training and a different understanding of one's own supervisory role (18). Nonetheless, the extent to which closed-loop modes under certain specific conditions (e.g., patient-ventilator asynchrony) are superior to the observation of conventional AOIs and the patients themselves is as yet unclear.

Ventilation curves had only low visual importance among the participants in the two groups, probably because ICU nurses are mainly trained to keep an eye on numbers in their professional formation. Another possible reason might be the higher degree of abstraction of ventilation curve shapes, leading to visual disregard. Further, it could be more difficult to cognitively draw conclusions about the patient's respiratory condition by fixating ventilation curves as compared to numeric values or to infer ventilation strategies from the shape of abstract ventilation curves.

This study illustrates that eye-tracking is a useful tool in measuring and quantifying the distribution of visual attention of critical care nurses using a closed-loop system and to reveal differences between inexperienced and experienced participants. Biases due to differences in nurses' workload were minimized, as it proved to be similar among participants.

A main strength of this study is its pragmatic, non-simulated, real-life design. Further, the long tracking time of ~1 h gives a realistic picture of the handling of the respirator, reflecting everyday situations in the ICU. To our knowledge, no comparable real-life studies in an ICU exist. Eye-tracking within such a framework might also assist in designing further novel and innovative studies.

The study has limitations. First, it was a single center study with probable biases due to the specific training of the nurses in I-ASV. Second, the participant number was relatively low. Nevertheless, we found comparable and homogenous distribution of data across dwell time, revisits, average fixation time, first fixation and fixation count among the participants, which adds to the credibility of the data. Third, the patients were from different medical fields, which might mean they had different pulmonary conditions, making distinct ventilation strategies necessary and leading to biases. Moreover, no specific study task was given to the participants, probably making comparisons more difficult. However, the study was explicitly designed to address ICU nurses' everyday behavior in their normal environment and the implementation of a specific task might itself have led to biases (e.g., awareness of the aim of the study). A further limitation of the eye-tracking technology is the difficulty of linking gaze patterns with cognition. Thus, the technology of eye-tracking should be seen as a complementary tool helping to objectively evaluate visual behavior and the visual interaction between humans and machines. This might provide further insights into the significance of visual situation awareness. Further studies with higher participant numbers are needed as well as randomized studies addressing similar questions in nurses with longer professional experience with closed-loop systems. Owing to the probable limitations of classic performance assessment and questionnaire-based human factors analyses in determining individual expertise on ventilation, a neuroscience approach with newer technologies such as eye-tracking could offer more objective and sensitive insights into human factors and human-machine interactions. As a consequence, eye-tracking might also contribute to improved patient safety, enhanced incidence reporting or the detection of factors leading to erroneous behavior in the ICU.

In conclusion, this study demonstrates that the visual fixations of nurses using I-ASV largely remained focused on traditional ventilation parameters. However, experienced nurses fixated AOIs related to the closed-loop system more often than did inexperienced ones, implying the need for constant training and education with new tools in critical care, especially for inexperienced professionals.

## Data Availability Statement

The raw data supporting the conclusions of this article will be made available by the authors, without undue reservation.

## Ethics Statement

The studies involving human participants were reviewed and approved by Kantonale Ethikkommission Zurich BASEC ID REQ 2017-00798. The patients/participants provided their written informed consent to participate in this study.

## Author Contributions

PB, AH, and DH conceived and designed the study, recruited the patients, collected the data, and drafted the report. PB did the ethics submission. PB, AH, NB, SK, SW, PW, MK, QL, ES, RS, and DH analyzed and interpreted the data and contributed to reviewing it. All authors read and approved the final manuscript.

## Conflict of Interest

The authors declare that the research was conducted in the absence of any commercial or financial relationships that could be construed as a potential conflict of interest.

## Publisher's Note

All claims expressed in this article are solely those of the authors and do not necessarily represent those of their affiliated organizations, or those of the publisher, the editors and the reviewers. Any product that may be evaluated in this article, or claim that may be made by its manufacturer, is not guaranteed or endorsed by the publisher.
