# Supplementary material for: Differing Visual Behavior Between Inexperienced and Experienced Critical Care Nurses While Using a Closed-Loop Ventilation System—A Prospective Observational Study
Source: Front Med (Lausanne). 2021 Sep 8;8:681321. doi: 10.3389/fmed.2021.681321 (PMC8455837; doi:10.3389/fmed.2021.681321)
Supplement: Supplementary file 1 [file Data_Sheet_1.docx]

Supplementary Material

**Supplementary Table 1.** Absolute values for Dwell time, Revisits, Average fixation, First fixation and Fixation count for all AOIs, corresponding to Figure 2. Numbers in () indicating interquartile range.

|  | **Area of interest AOI** | | | | |
| --- | --- | --- | --- | --- | --- |
|  | Settings | Ventilation curves | Numeric values | Oxygenation intellivent | Ventilation intellivent |
| Dwell time (ms) | 85330 (32989-170001) | 8022 (1759-24628) | 23813 (13477-63356) | 12563 (7190-32709) | 15917 (5347-36199) |
| Revisits (n) | 53 (17-78) | 11.5 (3.5-27.75) | 37 (20.75-92) | 21 (11-49) | 23 (6.5-34.5) |
| Average fixation (ms) | 5849 (2556-6964) | 664.8 (480.2-846.8) | 2793 (2132-3350) | 1242 (865.4-1710) | 1580 (1192-2336) |
| First fixation (ms) | 4913 (2274-7087) | 456 (381.6-647.6) | 2539 (1784-3656) | 1062 (659.6-1357) | 1535 (1061-1975) |
| Fixation count (n) | 262.5 (102-525) | 31 (10-78) | 76.5 (48.75-173.3) | 48.5 (29.5-111.3) | 54 (18-113.5) |

**Supplementary Table 2.** Multiple comparisons corresponding to Figure 2 analyzed by Friedman’s and Dunn’s multiple comparisons test. P-value < 0.05 considered statistically significant.

|  | **Dwell time** | **Revisits** | **Average fixation** | **First fixation** | **Fixation count** |
| --- | --- | --- | --- | --- | --- |
|  |  |  |  |  |  |
| **Multiple comparisons of AOIs** | adjusted p value | adjusted p value | adjusted p value | adjusted p value | adjusted p value |
| Ventilation curves vs. Settings | **<0.0001** | **<0.0001** | **<0.0001** | **<0.0001** | **<0.0001** |
| Numeric values vs. Settings | **0.0153** | >0.9999 | 0.5722 | 0.8317 | **0.0153** |
| Oxygenation intellivent vs. Settings | **<0.0001** | **0.0204** | **<0.0001** | **<0.0001** | **<0.0001** |
| Ventilation intellivent vs. Settings | **<0.0001** | **0.0114** | **0.0004** | **0.0012** | **<0.0001** |
| Numeric values vs. Ventilation curves | **0.0033** | **0.0001** | **<0.0001** | **<0.0001** | **0.002** |
| Oxygenation intellivent vs. Ventilation curves | 0.2511 | 0.2249 | **0.0464** | 0.0995 | 0.1425 |
| Ventilation intellivent vs. Ventilation curves | >0.9999 | 0.3461 | **0.0008** | **0.0012** | 0.8317 |
| Oxygenation intellivent vs. Numeric values | >0.9999 | 0.3461 | **0.0098** | **0.0072** | >0.9999 |
| Ventilation intellivent vs. Numeric values | 0.4252 | 0.2249 | 0.2799 | 0.3461 | 0.4702 |
| Ventilation intellivent vs. Oxygenation intellivent | >0.9999 | >0.9999 | >0.9999 | >0.9999 | >0.9999 |

**Supplementary Table 3.**

Group comparisons between inexperienced and experienced participants for all AOIs

analyzed by the Mann-Whitney’s test. P-value < 0.05 considered statistically significant.

| **Inexperienced vs. experienced group** | **p-value** |
| --- | --- |
| **according to AOI** |  |
|  |  |
| **Dwell Time (ms)** |  |
| Settings | 0.1212 |
| Ventilation curves | 0.3070 |
| Numeric values | 0.2803 |
| Oxygenation Intellivent | **0.0473** |
| Ventilation Intellivent | 0.1703 |
|  |  |
| **Revisits (n)** |  |
| Settings | **0.0144** |
| Ventilation curves | 0.1411 |
| Numeric values | 0.2267 |
| Oxygenation Intellivent | 0.0641 |
| Ventilation Intellivent | 0.0674 |
|  |  |
| **Average fixation (ms)** |  |
| Settings | **0.0071** |
| Ventilation curves | 0.4032 |
| Numeric values | 0.5369 |
| Oxygenation Intellivent | **0.0052** |
| Ventilation Intellivent | 0.2752 |
|  |  |
| **First fixation (ms)** |  |
| Settings | **0.0015** |
| Ventilation curves | 0.1019 |
| Numeric values | 0.3965 |
| Oxygenation Intellivent | **0.0197** |
| Ventilation Intellivent | 0.3410 |
|  |  |
| **Fixation Count (n)** |  |
| Settings | 0.0999 |
| Ventilation curves | 0.1926 |
| Numeric values | 0.3410 |
| Oxygenation Intellivent | 0.0500 |
| Ventilation Intellivent | 0.2361 |
